# Differential Analysis of Stress Tolerance and Transcriptome of Probiotic *Lacticaseibacillus casei* Zhang Produced from Solid-State (SSF-SW) and Liquid-State (LSF-MRS) Fermentations

**DOI:** 10.3390/microorganisms8111656

**Published:** 2020-10-26

**Authors:** Pengyu Wu, Jing An, Liang Chen, Qiuyan Zhu, Yingjun Li, Yuxia Mei, Zhenmin Chen, Yunxiang Liang

**Affiliations:** State Key Laboratory of Agricultural Microbiology, College of Life Science and Technology, Huazhong Agricultural University, Wuhan 430070, China; 18771068351@163.com (P.W.); 15271941472@163.com (J.A.); 13797068421@163.com (L.C.); 15575550652@163.com (Q.Z.); yingjun@mail.hzau.edu.cn (Y.L.); mei@mail.hzau.edu.cn (Y.M.); zmchen@mail.hzau.edu.cn (Z.C.)

**Keywords:** *Lacticaseibacillus casei* Zhang, solid-state fermentation, liquid-state fermentation, stress tolerance, RNA-seq

## Abstract

The property differences between bacteria produced from solid-state and liquid-state fermentations have always been the focus of attention. This study analyzed the stress tolerance and transcriptomic differences of the probiotic *Lacticaseibacillus casei* Zhang produced from solid-state and liquid-state fermentations under no direct stress. The total biomass of *L. casei* Zhang generated from liquid-state fermentation with MRS medium (LSF-MRS) was 2.24 times as much as that from solid-state fermentation with soybean meal-wheat bran (SSF-SW) medium. Interestingly, NaCl, H_2_O_2_, and ethanol stress tolerances and the survival rate after *L. casei* Zhang agent preparation from SSF-SW fermentation were significantly higher than those from LSF-MRS fermentation. The global transcriptomic analysis revealed that in *L. casei* Zhang produced from SSF-SW fermentation, carbohydrate transport, gluconeogenesis, inositol phosphate metabolism were promoted, that pentose phosphate pathway was up-regulated to produce more NADPH, that citrate transport and fermentation was extremely significantly promoted to produce pyruvate and ATP, and that pyruvate metabolism was widely up-regulated to form lactate, acetate, ethanol, and succinate from pyruvate and acetyl-CoA, whereas glycolysis was suppressed, and fatty acid biosynthesis was suppressed. Moreover, in response to adverse stresses, some genes encoding aquaporins (GlpF), superoxide dismutase (SOD), nitroreductase, iron homeostasis-related proteins, trehalose operon repressor TreR, alcohol dehydrogenase (ADH), and TetR/AcrR family transcriptional regulators were up-regulated in *L. casei* Zhang produced from SSF-SW fermentation. Our findings provide novel insight into the differences in growth performance, carbon and lipid metabolisms, and stress tolerance between *L. casei* Zhang from solid-state and liquid-state fermentations.

## 1. Introduction

Lactic acid bacteria (LAB) have been generally recognized as safe probiotics since they are native inhabitants of the oral cavity and the digestive tract of humans [1,2,3]. As the representative LAB, *L. casei* has been isolated from a variety of environmental habitats, including raw and fermented dairy and plant materials as well as the gastrointestinal tracts of humans and animals, and traditionally, this bacterium is recognized as probiotics and applied in commercial products for promoting nutrition and health [4,5,6]. The probiotic strain, *L. casei* Zhang, isolated from homemade koumiss in Inner Mongolia of China, has been commercially used as starter in the manufacture of dairy products [7,8,9,10]. To date, *L. casei* Zhang has been confirmed to have multiple functions including ameliorating the high-fructose-induced impaired glucose tolerance in hyperinsulinemia rats [11], affecting the gene expression in hypercholesterolemic rat liver to stimulate lipid metabolism [12], protecting against the endotoxin- and d-galactosamine-induced liver injury in rats [13], and preventing the intestinal tumorigenesis in mice [14]. In addition, in humans, *L. casei* Zhang was found to elevate the fecal short-chain fatty acid level, stabilize gut microbiota in adults, and decrease gut microbiota age index in older adults and the total colonic bile acid level [15,16,17]. However, similar to other LAB, *L. casei* Zhang often encounters various stresses both in food preparation, storage, and gastrointestinal tract, such as high/low temperature, acid and alkaline, high oxidation stress, high hydrostatic pressure and osmotic pressure, starvation, and antibiotics [18,19,20]. Therefore, the study of the stress response mechanism of *L. casei* Zhang is of great significance for improving its stress tolerance.

Nowadays, the responses of *L. casei* Zhang to such stresses as acid, antibody, bile salts, and high or low temperature have been studied. The carbohydrate metabolism of *L. casei* Zhang plays an important role under low acid stress [21]. The alterations in membrane fluidity, fatty acid distribution, and cell integrity are common strategies for *L. casei* Zhang to withstand severe acidification and to reduce the deleterious effect of lactic acid [22]. Further study reveals that the exogenous aspartate can improve the growth performance and acid tolerance of *L. casei* Zhang under acid stress [23]. *L. casei* Zhang has been found to adapt amoxicillin stress and gentamycin stress by activating the metabolism pathways of carbohydrate, amino acid, and purine, especially alkaline shock protein (ASP23) [24]. The proteins related to cell protection (DnaK and GroEL), cell membrane modifications (NagA, GalU, and PyrD) and key components in central metabolism (PFK, PGM, CysK, LuxS, PepC, and EF-Tu) are involved in a complex physiological response of *L. casei* Zhang under bile salts stress [25]. Mg^2+^, the second most abundant cation in bacteria, has been found to play a significant role in the thermotolerance of *L. casei* Zhang [26]. However, the response mechanisms of *L. casei* Zhang to other stresses including H_2_O_2_ and ethanol remain to be further investigated.

The commonly used medium for most LAB including *L. casei* is de Man–Rogosa–Sharp (MRS) medium which can be modified with nitrous acid, sorbitol, galactose, or maltose [27,28]. In addition, as a cost-efficient fermentation technology for LAB, solid-state fermentation (SSF) using soybean as substrate is employed to produce the soybean-fermented foods, such as sufu (a Chinese fermented soybean food), soybean flour, and soymilk [29,30]. Nowadays, the transcriptional analysis of *L. casei* Zhang directly exposed to various stresses have been extensively conducted. However, little research focuses on the differences in stress-tolerance and transcriptome between *L. casei* Zhang produced from solid-state fermentation and liquid-state fermentation under no direct stress. Therefore, in this study, the differences in the transcriptome level and the tolerance of *L. casei* Zhang from two different fermentations to NaCl, H_2_O_2_, and ethanol stresses were investigated.

## 2. Materials and Methods

### 2.1. Bacterial Strain, Media, and Growth Conditions

*L. casei* Zhang wild-type strain was obtained from the School of Food Science and Engineering, Inner Mongolia Agricultural University, China. Liquid-state fermentation de Man–Rogosa–Sharp (LSF-MRS) medium contained 10.0 g/L of peptone, 8.0 g/L of beef extract powder, 4.0 g/L of yeast extract powder, 20.0 g/L of glucose, 0.2 g/L of MgSO_4_·7H_2_O, 5.0 g/L of sodium acetate, 2.0 g/L of sodium citrate, 2.0 g/L of K_2_HPO_4_, 0.15 g/L of MnSO_4_, and 1.0 mL of Tween-80. Solid-state fermentation (SSF-SW) medium contained 60.0 g soybean meal, 20.0 g wheat bran, and 48.0 mL deionized water. *L. casei* Zhang from a −80 °C glycerol stock was streaked onto MRS agar plate and cultured for 24 h at 37 °C. A single colony of *L. casei* Zhang was incubated into 5.0 mL MRS medium for overnight culture at 37 °C for further experiments.

### 2.2. Preparation of L. casei Zhang Bacterial Suspensions after Solid-State and Liquid-State Fermentation

The overnight cultured *L. casei* Zhang was inoculated into 128.0 g fresh SSF-SW and 400.0 mL LSF–MRS media with 1.0% inoculum size and cultured for 14 h at 37 °C.

For bacterial collection from SSF-SW medium, 400.0 mL of 0.85% sterilized saline water was added into the fermentation system and shaken at 200× *g* for 5 min at 37 °C. Then the mixture was filtrated by 2-layer gauze and the filtrate was centrifuged at 5000× *g* for 5 min. The obtained precipitate was washed twice and finally resuspended with 400.0 mL of sterilized 0.85% saline water. For bacterial collection from LSF-MRS medium, fermentation liquid was centrifuged at 5000× *g* for 5 min. The obtained precipitate was washed twice and finally resuspended with 400.0 mL of sterilized 0.85% saline water. The concentration of bacterial suspension was calculated by spread plate method with the following formula.

Total biomass after fermentation (CFU) = Concentration of bacterial suspension (CFU/mL) × resuspension volume (mL).

### 2.3. Sensitivity of L. casei Zhang to NaCl, H_2_O_2_, and Ethanol after Solid-State and Liquid-State Fermentations

The OD_600 nm_ of bacterial suspensions was adjusted to 1.50, and the initial bacterial count was calculated by spread plate method. In NaCl stress test, the bacterial suspensions were centrifuged at 5000× *g* for 5 min and resuspended with 15.0%, 20.0%, and saturated (at 30 °C) NaCl solutions. After the resultant bacterial resuspensions were placed at 30 °C for 10 min, the bacterial count was calculated by spread plate method. In H_2_O_2_ stress test, the bacterial suspensions were centrifuged at 5000× *g* for 5 min and resuspended with sterilized 0.85% saline water containing 0.40%, 0.6%, 0.8%, and 1.0% H_2_O_2_, respectively. After the obtained bacterial resuspensions were placed at 30 °C for 10 min, the bacterial count was calculated by spread plate method. In ethanol stress test, the bacterial suspensions were centrifuged at 5000× *g* for 5 min and resuspended with the sterilized 0.85% saline water containing 10%, 20%, and 30% ethanol, respectively. After the resuspensions were placed at 30 °C for 10 min, the bacterial count was calculated by spread plate method.

Bacterial survival rate (%) = (initial bacterial count − bacterial count after stress treatment)/initial bacterial count × 100%.

### 2.4. Bacterial Agent Preparation by Cold-Air Drying and Spray Drying

The concentration (*C*) of bacterial suspensions was determined by gradient dilution counting. For cold-air drying, the sterilized wheat bran with 0.8 times bacterial suspension weight was added into the pre-prepared bacterial suspensions. After being mixed uniformly, the bacterial suspensions were dried for 6 h at 30 °C and 10% humidity with wind gear 8–9 on air drying machine YCFZD-2A (Ouyi Electric Appliance co. LTD, Hangzhou, China). The resultant mixture was stirred once every hour to prevent the bacterial agents from hardening. Finally, weight (*m*) and biomass (*b*) of bacterial agents were determined and the survival rates (*S*) after bacterial agent preparation were calculated.

$$\mathrm{Survival}\;\mathrm{rate}\;\left(\%\right)\;S\;=\;\frac{\;m\times b}{\;C\times V}\times 100\%$$

where *C* is concentration of the bacterial suspensions (CFU/mL); *V* is the volume of the bacterial suspensions (mL); *m* is weight of the bacterial agents after cold-air drying (g); *b* is biomass of bacterial agents after cold-air drying (CFU/g).

For spray drying, the bacterial suspensions were centrifuged at 5000× *g* for 5 min and resuspended with sterilized water. Then the skimmed milk powder with 0.2 times bacterial suspension weight was added into the pre-prepared bacterial suspensions. Concentrations of these bacterial skimmed milk suspensions were determined by spread plate method. After being mixed uniformly, the suspensions were dried at a constant air inlet temperature of 140 °C and air outlet temperature of 60 °C on spray dryer YC-015 (Yacheng Instrument & Equipment co. LTD, Shanghai, China). Finally, biomass (*b*) of the bacterial agents were determined and the survival rates (*S*) after spray drying were calculated.

$$\mathrm{Survival}\;\mathrm{rate}\;\left(\%\right)\;S\;=\;\frac{\;b\times m}{\;B\times V}\times 100\%$$

where *b* is biomass of the bacterial agents after spraydrying (CFU/g); *m* is the total weight of the spray-dried powder (g); *B* is biomass of bacterial skimmed milk suspension (CFU/mL). *V* is total volume of the bacterial skimmed milk suspension used for spray drying (mL).

### 2.5. RNA Extraction, RNA-Seq, Transcriptomic Data Processing, and RNA-Seq Data Accession Number

RNA extraction, RNA-seq, and transcriptomic data processing were accomplished by BGI Technology Services Co., LTD (Shenzhen, China).

The bacterial cells were collected through centrifugation at 5000× *g* and 4 °C for 10 min. The cell precipitates were flash-frozen in liquid nitrogen and then treated with trizol at −80 °C. Total RNA was isolated by using TRIzol reagent (Invitrogen, Waltham, MA, USA) and purified by using the Rio-Zero rRNA Removal Kit (Illumina, San Diego, CA, USA) according to the manufacturer’s instructions. Degradation and contamination of the as-prepared RNA was monitored on 1.5% agarose gels. Then, RNA concentration was measured with Qubit^®^ RNA Assay Kit in Qubit^®^ 3.0 Flurometer (Life Technologies, Carlsbad, CA, USA). RNA integrity was assessed with the RNA Nano 6000 Assay Kit in Agilent 2100 Bioanalyzer system (Agilent Technologies, Santa Clara, CA, USA) (RIN > 9.0). Every experimental group set three independent biological replicates in this study.

A total amount of 3 μg RNA per sample was used as input material for the RNA sample preparations. The RNA samples were added into Fragmentation Buffer to conduct PCR for thermal fragmentation into 130–160 nt. First-strand cDNA was generated by First Strand Mix, and second-strand cDNA was generated by Second Strand Mix. Afterwards, the purified fragmented cDNA was combined with End Repair Mix and incubated with A-Tailing Mix for adding “A” to the end of the cDNA. Then, the resultant Adenylate 3’Ends DNA was incubated with RNA Index Adapter and Ligation Mix to be linked to adapter. PCR amplification was performed with PCR Mix to enrich the cDNA fragments, and then the PCR products were purified with Ampure XP Beads (AGENCOURT, Beverly, MA, USA). Library was validated with the Technologies 2100 bioanalyzer (Agilent, Santa Clara, CA, USA) for quality control. The double-stranded PCR products were heat denatured and circularized by the splint oligo sequence. The single-stranded circle DNA (ssCir DNA) was formatted as the final library. The final library was amplified with phi29 (Thermo Fisher Scientific, Waltham, MA, USA) to make DNA nanoball (DNB), and in this final library, every molecule had more than 300 copies. DNBs were loaded into the patterned nanoarray, and single-end 50-base reads were generated on BGISEQ500 platform (BGI, Shenzhen, China).

The sequencing raw data were filtered by SOAPnuke v1.5.2 [31] to remove the reads with low quality, reads with adapter pollution, and reads with unknown base (N) (content greater than 10%), and then these processed data were saved as FASTQ format. All the clean data were aligned to the genome of *L. casei* Zhang (firmicutes) (GenBank Accession No. NC_014334.2 and NC_011352.1). Then, these clean data were aligned to the reference gene sequence by Bowtie2 [32], and then the expression levels of genes and transcripts were calculated by RSEM [33]. Differential expression (DE) analysis was performed by using the DEseq2 (Fold Change >= 2 and Adjusted *p*-value <= 0.001) [34]. The genes with *p*-value < 0.001 (adjusted in the Benjamini and Hochberg’s approach) and fold change >2.0 were defined as differentially expressed. The differentially expressed genes (DEGs) were classified and enriched by Gene Ontology (GO) and KEGG PATHWAY annotation.

RNA-seq data were deposited in Sequence Read Archive under the accession numbers SRR12378022 (SSF-SW-1), SRR12378030 (SSF-SW-2), SRR12378029 (SSF-SW-3), SRR12378025 (LSF-MRS-1), SRR12378024 (LSF-MRS-2), and SRR12378023 (LSF-MRS-3).

### 2.6. Determination of Organic Acids

For *L. casei* Zhang fermented with LSF-MRS medium, samples (2.0 mL) were centrifuged at 5000× *g* for 10 min to obtain cell-free cultured supernatants. The supernatants were diluted 10 times and then filtered through disposable syringe filters (Millipore, 0.22 μm). For *L. casei* Zhang fermented with SSF-SW medium, samples (10.0 g) were added into 90.0 mL of 5 mM H_2_SO_4_ solution and then mixed in shaker at 180 rpm for 20 min. The mixture was centrifuged at 5000× *g* for 10 min to obtain cell-free cultured supernatants. The supernatants were also diluted 10 times and then filtered through disposable syringe filters (Millipore, 0.22 μm). Finally, 20 μL of the obtained solutions were analyzed by HPLC (LC-20A, Shimadzu, Japan) on a Bio-Rad HPX-87H ion-exclusion column (300 × 7.8 mm). Organic acids (lactate and acetate) were detected by a differential refraction detector and mobile phase was 5 mM H_2_SO_4_ that was pumped through the column at a flow rate of 0.6 mL/min at column temperature 40 °C. The peak area was used to calculate the concentrations of lactate and acetate according to corresponding standard curves.

### 2.7. Statistical Analysis

All presented data were the average of at least three biological replicates. Statistical analysis was carried out using SPSS 19.0 software. The statistically significant difference was determined using the Student’s *t*-test. *p*-value < 0.05 was considered as statistically significant.

## 3. Results and Discussions

### 3.1. Total Biomasses of L. casei Zhang after Fermentation with SSF-SW and LSF-MRS Media and Survival Rates of L. casei Zhang after Cold-Air Drying and Spray Drying

**Figure 1:**
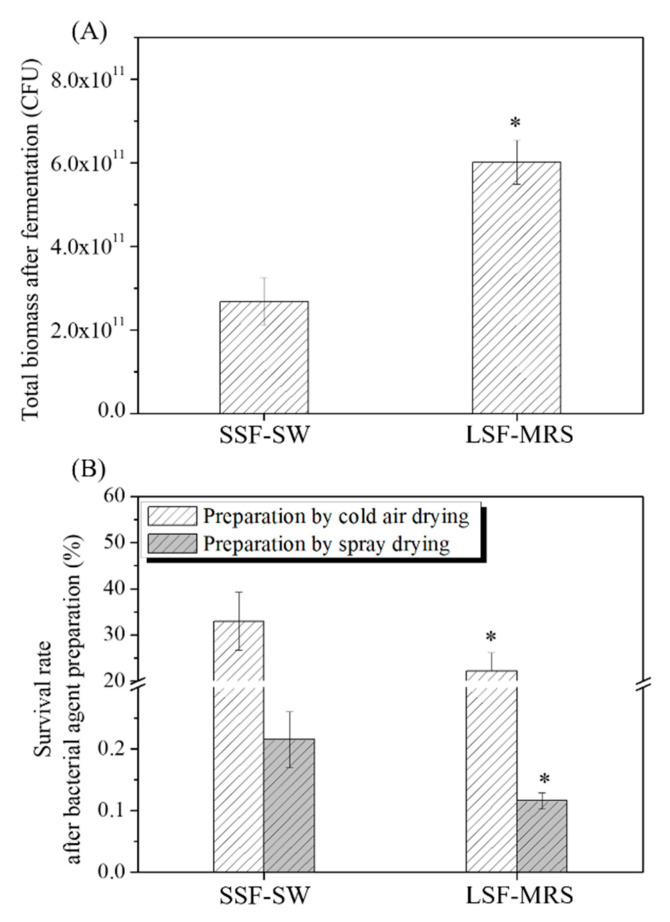
Total biomasses of *L. casei* Zhang after fermentation with solid-state fermentation (in SSF-SW medium) and liquid-state fermentation (in LSF-MRS medium) (**A**) and survival rates of *L. casei* Zhang after cold-air drying and spray drying (**B**). * Statistically significant differences were determined using Student’s *t*-test.

The effects of the SSF-SW and LSF-MRS fermentations on the total biomass of *L. casei* Zhang and survival rates of *L. casei* Zhang after cold-air drying and spray drying were investigated (Figure 1). The total biomass of *L. casei* Zhang fermented with LSF-MRS medium was found to be 6.01 × 10^11^ CFU, which was significantly higher than (2.24 times) that fermented with SSF-SW medium (2.68 × 10^11^ CFU) (*p*-value < 0.05) (Figure 1A). The differences in total biomass may be due to the lower level of nutrient substance in SSF-SW medium than in LSF-MRS medium. Surprisingly, in the bacterial agents after cold-air drying, the survival rate of *L. casei* Zhang fermented with LSF-MRS medium (22.13 ± 4.02%) was significantly lower than that fermented with SSF-SW medium (32.93 ± 6.25%) (*p*-value < 0.05) (Figure 1B). Similarly, in the bacterial agents after spray drying, the survival rate of *L. casei* Zhang fermented with LSF-MRS medium (0.12 ± 0.01%) was significantly lower than that fermented with SSF-SW medium (0.21 ± 0.05%) (*p*-value < 0.05) (Figure 1B). In order to examine the survival rate difference, the NaCl-, H_2_O_2_-, and ethanol- stress tolerances of *L. casei* Zhang fermented respectively with SSF-SW medium and LSF-MRS medium were determined.

### 3.2. Effects of NaCl, H_2_O_2_, and Ethanol Stresses on the Survival Rates of L. casei Zhang after Fermentation with SSF-SW and LSF-MRS Media

**Figure 2:**
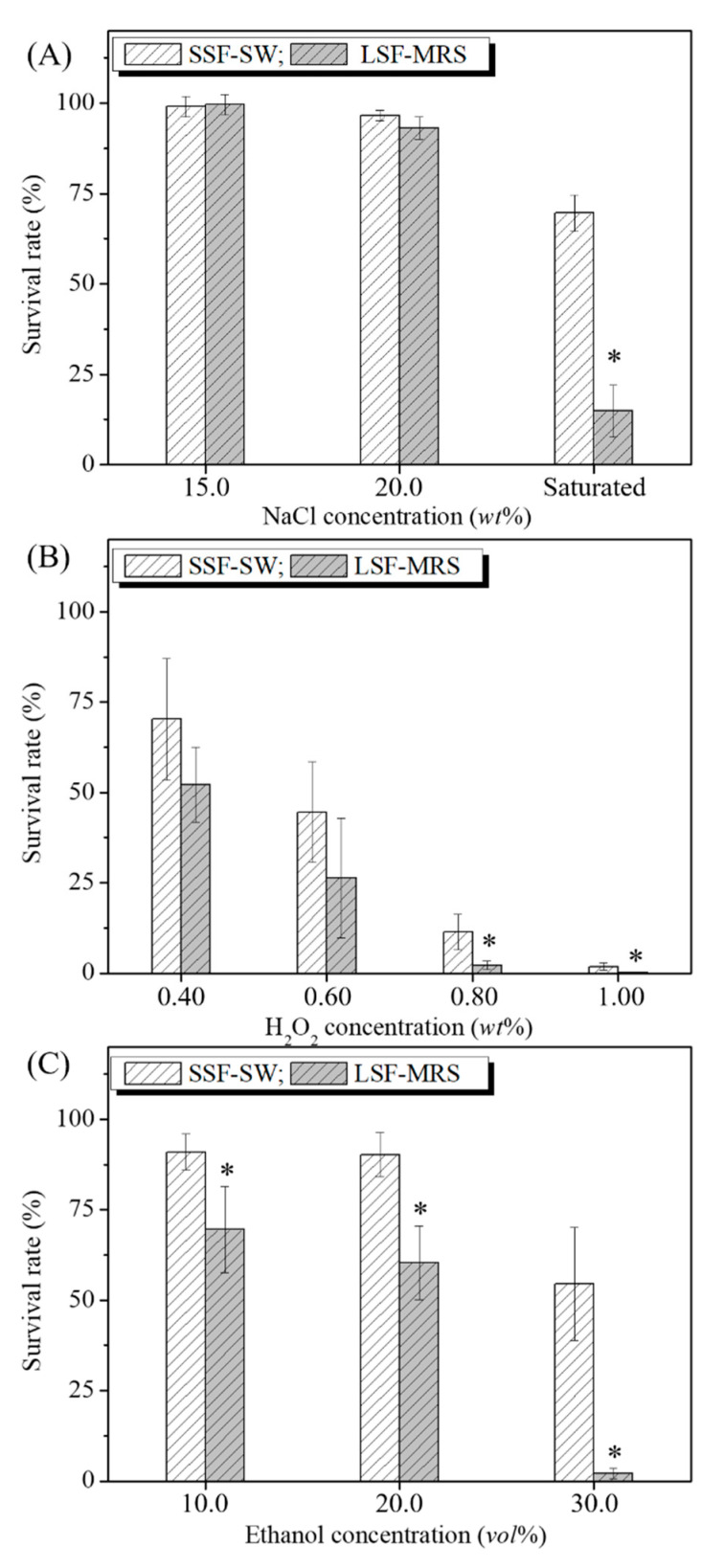
Effects of NaCl (**A**), H_2_O_2_ (**B**), and ethanol (**C**) on survival rates of *L. casei* Zhang after fermentation with solid-state fermentation (in SSF-SW medium) and liquid-state fermentation (in LSF-MRS medium). * Statistically significant differences were determined using Student’s *t*-test.

To test the sensitivity of *L. casei* Zhang after different fermentations to NaCl, H_2_O_2_, and ethanol, the survival rates of bacterial suspensions after fermentation with SSF-SW and LSF-MRS media were detected (Figure 2). Under the treatments respectively with 15.0%, 20.0%, and saturated NaCl (wt %), the survival rates of *L. casei* Zhang after fermentation with SSF-SW medium were 99.06 ± 2.89%, 96.55 ± 1.46%, and 69.58 ± 4.94%, whereas those of *L. casei* Zhang after fermentation with LSF-MRS medium were 99.64 ± 2.83%, 93.17 ± 3.06%, and 14.89 ± 7.14%, respectively. Under the treatment with saturated NaCl, the survival rate of *L. casei* Zhang after the fermentation with SSF-SW medium was significantly higher (4.67 times) than that after the fermentation with LSF-MRS medium (*p*-value < 0.05) (Figure 2A). Under the treatments respectively with 0.40%, 0.60%, 0.80% and 1.00% of H_2_O_2_, the survival rates of *L. casei* Zhang after fermentation with SSF-SW medium were 70.28 ± 16.76%, 44.57 ± 13.98%, 11.42 ± 4.86%, and 1.82 ± 0.97%, whereas those after fermentation with LSF-MRS medium were 52.20 ± 10.33%, 26.36 ± 16.54%, 2.17 ± 1.17%, and 0.13 ± 0.01%, respectively. Under the treatments with 0.80% and 1.00% of H_2_O_2_ (wt %), the survival rates of *L. casei* Zhang after fermentation with SSF-SW medium were significantly higher (5.26 times and 14.00 times) than those after fermentation with LSF-MRS medium (*p*-value < 0.05) (Figure 2B). Under the treatments with 10.0%, 20.0%, and 30.0% of ethanol (vol%), the survival rates of *L. casei* Zhang after fermentation with SSF-SW medium were 90.99 ± 5.08%, 90.22 ± 6.08%, and 54.56 ± 15.67%, whereas those after fermentation with LSF-MRS medium were 69.55 ± 11.89%, 60.37 ± 10.16%, and 2.14 ± 1.50%, respectively. Under the treatments with 10.0%, 20.0%, and 30.0% of ethanol (vol%), the survival rates of *L. casei* Zhang after fermentation with SSF-SW medium were significantly higher (1.31 times, 1.49 times, and 25.50 times) than those after fermentation with LSF-MRS medium (*p*-value < 0.05) (Figure 2C). Taken together, NaCl-, H_2_O_2_-, and ethanol- stress tolerances of *L. casei* Zhang after fermentation with SSF-SW medium were higher than those after fermentation with LSF-MRS medium.

### 3.3. Overview of L. casei Zhang Transcriptomic Response to Fermentation with SSF-SW and LSF-MRS Media

**Figure 3:**
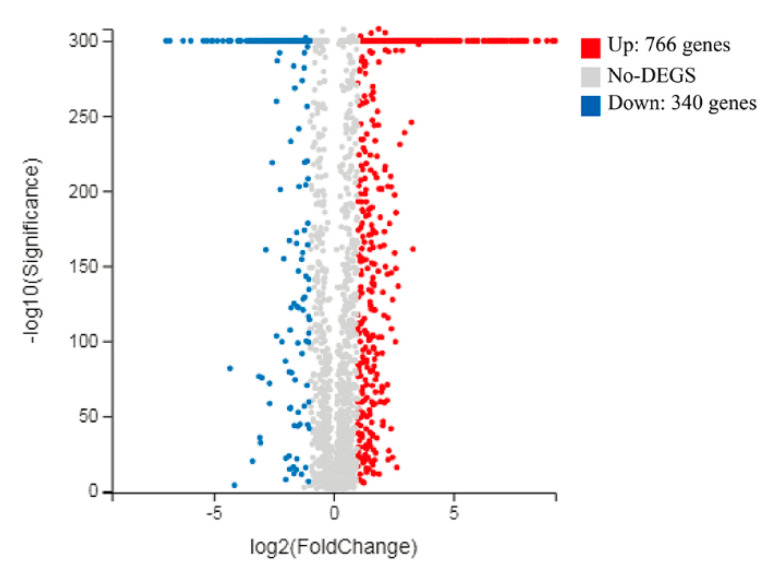
Volcano plot of DEGs between *L. casei* Zhang after fermentation with solid-state fermentation (in SSF-SW medium) and liquid-state fermentation (in LSF-MRS medium). Every circle point indicates one gene of *L. casei* Zhang. Fold change > 2.0, *p*-value < 0.001 are highlighted by blue and green lines, respectively. The red points indicate significant up-regulation and blue points represent significant down-regulation.

**Figure 4:**
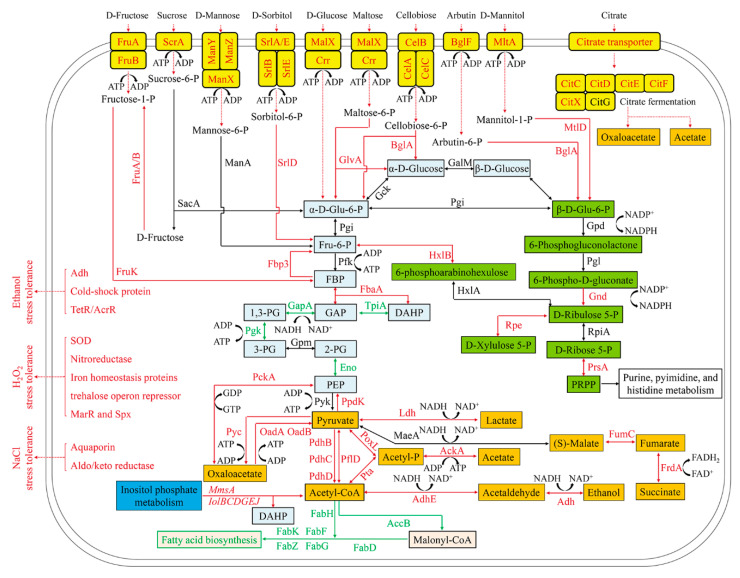
Schematic diagram of carbohydrate transport and metabolism differences and NaCl, H_2_O_2_, and ethanol stress tolerance differences of *L. casei* Zhang after fermentation with solid-state fermentation (in SSF-SW medium) and liquid-state fermentation (in LSF-MRS medium). Glu-6-P, glucose-6-phosphate; Fru-6-P, fructose-6-phosphate; FBP, fructose-1,6-diphosphate; DHAP, dihydroxyacetone-phosphate; GAP, glyceraldehyde-3-phosphate; 1,3-PG, 1,3-diphosphoglycerate; 3-PG, 3-phosphoglycerate; 2-PG, 2-phosphoglycerate; PEP, phosphoenolpyruvate. Red color stands for up-regulation; black color represents no change; green color indicates down-regulation at mRNA level.

The BGISEQ-500 platform was used to investigate the transcriptome level changes of *L. casei* Zhang after fermentation with solid-state fermentation (in SSF-SW medium) and liquid-state fermentations (in LSF-MRS media). The numbers of clean reads before and after trimming are shown in Table S1. The information on reads mapping to the reference genome and genes are shown in Table S2. Pearson correlation analysis was performed to investigate correlation of gene expression levels between the independent biological replicates (Figure S1). In addition, principal component analysis (PCA) showed that gene expression levels exhibited high similarities among three independent biological replicates within a certain group, whereas great differences were observed between the different groups (Figure S2). As shown in Figure 3, differential expression analysis through volcano plot (fold change > 2.0, *p*-value < 0.001) showed that a total of 1106 differentially expressed genes (DEGs) were identified, which consisted of 340 up-regulated genes and 766 down-regulated genes (SSF-SW vs. LSF-MRS). Those genes with different putative functions were classified into different categories by the gene ontology (GO) (Figure S3) and KEGG Pathway analyses (Figure S4). The DEGs involved in various biological processes such as carbohydrate transport, carbohydrate metabolism (including glycolysis/gluconeogenesis, pentose phosphate pathway, inositol phosphate metabolism, and pyruvate metabolism), and fatty acid metabolism, and the DEGs related to NaCl-, H_2_O_2_-, and ethanol- stress tolerances and some hypothetical proteins are shown in Table S3 and Figure 4.

### 3.4. Enhancement of Carbohydrate Transport in L. casei Zhang Fermented with SSF-SW Medium

Two genes *(malX* and *crr*) belonging to maltose/glucose-specific phosphotransferase system (PTS) in *L. casei* Zhang fermented with SSF-SW medium were respectively 59.23-fold and 6.39-fold up-regulated, relative to these two genes in *L. casei* Zhang fermented with LSF-MRS medium. Maltose/glucose-specific PTS was responsible for transforming the extracellular D-glucose and maltose into the intracellular α-D-Glu-6-P and maltose-6-P. Meanwhile, gene *glvA* encoding maltose-6’-phosphate glucosidase that could further catalyze maltose-6-P to form α-D-Glu-6-P and D-glucose was 25.75-fold up-regulated. Ten genes (4 *manX*, 3 *manY*, and 3 *manZ*) involved in mannose-specific PTS responsible for transforming the extracellular D-mannose into the intracellular mannose-6-P were 2.38-fold to 198.44-fold up-regulated. Mannose-6-P can be further catalyzed by the *manA* gene encoding mannose-6-phosphate isomerase to form Fru-6-P. Three genes (*srlA*, *srlB*, and *srlE*) involved in glucitol/sorbitol-specific PTS responsible for transforming the extracellular D-sorbitol into the intracellular sorbitol-6-P were 9.40-fold, 13.08-fold, and 16.21-fold up-regulated, respectively. Two *srlD* genes encoding sorbitol-6-phosphate 2-dehydrogenase that could further catalyze sorbitol-6-P to form Fru-6-P were 4.61-fold and 9.94-fold up-regulated, respectively. Four genes (2 *fruA* and 2 *fruB*) involved in fructose-specific PTS which can transform the extracellular D-fructose into intracellular fructose-1-P exhibited 8.41-fold to 12.96-fold up-regulation. Two *fruK* genes encoding 1-phosphofructokinase that could further catalyze fructose-1-P to form FBP displayed 2.26- and 5.19-fold up-regulation. Twelve genes (4 *celA*, 4 *celB*, and 4 *celC*) involved in cellobiose-specific PTS which could transform the extracellular cellobiose into the intracellular cellobiose-P exhibited 2.24- to 6.00-fold up-regulation. Four *bglA* genes encoding 6-phospho-beta-glucosidase responsible for further catalyzing cellobiose-P to form α-D-Glu-6-P and α-D-Glucose were up-regulated 2.60- to 102.00-fold. One *scrA* gene involved in sucrose-specific PTS capable of transforming the extracellular sucrose into the intracellular sucrose-6-P was 3.72-fold up-regulated. The *sacA* gene could encode beta-fructofuranosidase that can further catalyze sucrose-6-P to form α-D-Glu-6-P and D-fructose. Furthermore, D-fructose could form D-fructose-1-P and further form FBP through dual phosphorylation. Three *mtlA* genes involved in mannitol-specific PTS capable of transforming the extracellular mannitol into the intracellular mannitol-1-P exhibited 2.20- to 3.40-fold up-regulation. The *mtlD* encoding mannitol-1-phosphate 5-dehydrogenase which can further catalyze mannitol-1-P to form β-D-Glu-6-P displayed 2.01-fold up-regulation. Two *bglF* genes involved in beta-glucoside-specific PTS responsible for transforming the extracellular arbutin (β-glucoside) into the intracellular arbutin-6-P were 31.95-fold and 240.76-fold up-regulated, respectively. Four *bglA* genes encoding 6-phospho-beta-glucosidase which could further catalyze arbutin-6-P to form β-D-Glu-6-P were 2.60- to 102.00-fold up-regulated. Therefore, in *L. casei* Zhang fermented with SSF-SW medium, the transport of D-fructose, sucrose, D-mannose, D-sorbitol, D-glucose, maltose, cellobiose, arbutin, and D-mannitol into glycolysis, gluconeogenesis, and pentose phosphate pathways were promoted, which conferred this strain a competitive survival advantage.

One citrate transporter gene in *L. casei* Zhang fermented with SSF-SW medium was 608.04-fold up-regulated, compared with that fermented with LSF-MRS medium. In addition, In *L. casei* Zhang fermented with SSF-SW medium, five genes (*citC*, *citD*, *citE*, *citF*, and *citX*) encoding a citrate lyase and involved in two-component system (citrate fermentation) that can catalyze citrate to form oxaloacetate and acetate [35] exhibited 213.04-, 167.88-, 136.28-, 101.75-, and 79.40-fold up-regulation, respectively.

### 3.5. Enhancement of Gluconeogenesis and Suppression of Glycolysis in L. casei Zhang after Fermentation with SSF-SW Medium

Two *fbaA* genes encoding fructose-bisphosphate aldolase which could catalyze the reversible reaction between FBP and DAHP or GAP, and one *fbp3* gene encoding fructose-1,6-bisphosphatase which could catalyze FBP to form Fru-6-P exhibited 2.76- to 5.01-fold up-regulation. However, gene *tpiA* encoding triosephosphate isomerase which could catalyze the reversible reaction between DAHP with GAP, gene *gapA* encoding glyceraldehyde 3-phosphate dehydrogenase which could catalyze the reversible reaction between GAP and 1,3-PG, gene *pgk* encoding phosphoglycerate kinase capable of catalyzing the reversible reaction between 1,3-PG and 3-PG, and two *eno* genes encoding enolase responsible for catalyzing the reversible reaction between 2-PG and PEP were found to be 2.07-fold to 5.74-fold down-regulated. Therefore, glycolysis was suppressed, but partial gluconeogenesis was enhanced in *L. casei* Zhang cultured with SSF-SW medium, compared with *L. casei* Zhang cultured with LSF-MRS medium. As a result, accumulation of α-D-Glu-6-P and Fru-6-P enhanced pentose phosphate pathway to produce more NADPH. This metabolic regulation could be a response to a starvation condition. Like *L. casei* differentially regulated enzymes relate to glycolysis under lactose starvation [36], *L. casei* Zhang enhanced the transport of a variety of carbohydrates, expanded the carbon source spectrum, and enhanced gluconeogenesis to make the use of various substances in cells more flexible to deal with starvation stress.

### 3.6. Enhancement of Pentose Phosphate Pathway in L. casei Zhang Cultured with SSF-SW Medium

Gene *gnd* encoding 6-phosphogluconate dehydrogenase capable of catalyzing 6-phospho-D-gluconate to form D-ribulose 5-P and further produce NADPH, gene *hxlB* encoding 6-phospho-3-hexuloisomerase, gene *hxlA* encoding 6-phosphoarabinohexulose which could convert Fru-6-P into 6-phosphoarabinohexulose and further into D-Ribulose 5-P, gene *rpe* encoding ribulose-phosphate 3-epimerase capable of catalyzing the reversible reaction between D-ribulose 5-P and D-xylulose 5-P, and gene *prsA* encoding ribose-phosphate pyrophosphokinase responsible for catalyzing the reversible reaction between D-Ribose 5-P and PRPP were 2.38- to 5.11-fold up-regulated. Therefore, relative to that in *L. casei* Zhang cultured with LSF-MRS medium, pentose phosphate pathway in *L. casei* Zhang cultured with SSF-SW medium was enhanced to produce more NADPH which can further provide reducing power for stress response [37,38].

### 3.7. Enhancement of Inositol Phosphate Metabolism and Suppression of Fatty Acid Synthesis in L. casei Zhang Cultured with SSF-SW Medium

Eight genes (*mmsA*, *iolB*, *iolC*, *iolD*, two *iolG*, *iolE*, and *iolJ*) related to inositol phosphate metabolism were 3.03- to 9.91-fold up-regulated, which could convert (+)-inositol and myo-inositol into acetyl-CoA and DAHP. Meanwhile, in *L. casei* Zhang cultured with SSF-SW medium, fatty acid metabolism-related gene *accB* encoding acetyl-CoA carboxylase biotin carboxyl carrier protein which could catalyze acetyl-CoA to form malonyl-CoA was 3.06-fold down-regulated. In addition, fatty acid biosynthesis-related seven genes (*fabF*, *fabG*, *fabD*, *fabK*, *fabH*, and two *fabZ*) were also found to be down-regulated (2.19- to 4.90-fold). Taken together, inositol phosphate metabolism was enhanced, but fatty acid synthesis was suppressed so that acetyl-CoA was accumulated and the further relating metabolism based on acetyl-CoA might be stimulated in *L. casei* Zhang cultured with SSF-SW medium.

### 3.8. Enhancement of Pyruvate Metabolism in L. casei Zhang Cultured with SSF-SW Medium

Two genes (*oadA* and *oadB*) encoding the subunits of oxaloacetate decarboxylase were 58.50-fold, and 345.19-fold up-regulated, respectively, and this oxaloacetate decarboxylase could convert oxaloacetate into pyruvate, thus producing ATP. However, gene *pyc* encoding pyruvate carboxylase which could convert pyruvate into oxaloacetate and consume ATP was only 2.03-fold up-regulated. Meanwhile, gene *pckA* encoding phosphoenolpyruvate carboxykinase which could catalyze the reversible reaction between oxaloacetate and PEP was 2.08-fold up-regulated. The PEP could be further converted into pyruvate by gene *pyk* encoding pyruvate kinase to form ATP. Correspondingly, gene *ppdK* encoding pyruvate orthophosphate dikinase was 3.10-fold up-regulated, and this enzyme could convert pyruvate into PEP and consume ATP. As a result, in *L. casei* Zhang cultured with SSF-SW medium, pyruvate formation from oxaloacetate was enhanced.

Genes *ldh* and *ldhA* were 5.59-fold and 3.02-fold up-regulated, and these two genes were responsible for encoding L-lactate dehydrogenase and D-lactate dehydrogenase to catalyze the conversion from pyruvate into lactate. Meanwhile, genes *maeA*, *fumC*, and *frdA* were respectively 0-fold, 2.23-fold, and 2.08-fold up-regulated, and these three genes were responsible for encoding malate oxidoreductase, fumarate hydratase, and fumarate reductase flavoprotein subunit to convert pyruvate into (S)-malate, fumarate, and succinate. Three *pdhBCD* genes were 2.02-fold, 2.26-fold, and 2.54-fold up-regulated, respectively, and these three *pdhBCD* genes encoded the subunits of pyruvate dehydrogenase to convert pyruvate into acetyl-CoA. Meanwhile, gene *pflD* encoding formate C-acetyltransferase was 38.86-fold up-regulated, and this formate C-acetyltransferase catalyzed the reversible reaction between pyruvate and acetyl-CoA. Overall, the generation of lactate, succinate, and acetyl-CoA from pyruvate in *L. casei* Zhang after fermentation with SSF-SW medium was enhanced. Furthermore, gene *adhE* encoding acetaldehyde dehydrogenase/alcohol dehydrogenase and gene *adh* encoding alcohol dehydrogenase were 2.27-fold and 2.95-fold up-regulated, respectively, and these gene-encoded enzymes catalyzed the conversion from acetyl-CoA into acetaldehyde, and further into ethanol.

Gene *poxL* encoding pyruvate oxidase and gene *pta* encoding phosphate acetyltransferase were respectively 6.74-fold and 5.63-fold up-regulated, and these two enzymes catalyzed the conversion from pyruvate and acetyl-CoA into acetyl-P. Meanwhile, gene *ackA* encoding acetate kinase was 4.16-fold up-regulated, and acetate kinase converted acetyl-P into acetate, further producing ATP. Since glycolysis was suppressed in *L. casei* Zhang fermented with SSF-SW medium, ATP compensatory mechanism probably lay in conversion from oxaloacetate into pyruvate and conversion from acetyl-P into acetate, which was different with ATP formation from glycolysis in *Lactobacillus plantarum* CAUH2 in response to hydrogen peroxide stress [37].

### 3.9. Up-Regulation of Stress-Tolerance genes in L. casei Zhang Cultured with SSF-SW Medium

Relative to the genes in *L. casei* Zhang cultured with LSF-MRS medium, in *L. casei* Zhang cultured with SSF-SW medium, two genes which encoded aquaporins (GlpF) and aldo/keto reductase related to NaCl stress-tolerance were 30.02-fold and 2.34-fold up-regulated, respectively. As the transmembrane channel proteins, aquaporins exhibited 10- to 100-fold up-regulation in *L. plantarum,* and aquaporins were responsible for the transfer of water molecule, glycerol, and lactic acid through the cell membrane to maintain cellular osmotic pressure in bacteria and plant [39,40]. In addition, previous study demonstrated that the aldo/keto reductase-1 (AKR1) protected cellular enzymes from salt stress by detoxifying reactive cytotoxic compounds [41].

The H_2_O_2_ stress tolerance-related genes which encoded iron homeostasis proteins, nitroreductase, superoxide dismutase, transcriptional regulator Spx, MarR family transcriptional regulator, and trehalose operon repressor TreR were found to be 2.27-fold to 94.03-fold up-regulated in *L. casei* Zhang cultured with SSF-SW medium, relative to those cultured with LSF-MRS medium. *L. plantarum* CAUH2 [37] and *Giardia intestinalis* [42] made response to oxidative stress by enhancing expression of NADH peroxidase, thiol peroxidase, thioredoxin, glutathione peroxidase, and glutathione reductase. However, relative to those cultured with LSF-MRS medium, only two genes encoding nitroreductase (one of O_2_-consuming nitric oxide detoxification enzymes) and superoxide dismutase (SOD) (one of reactive oxygen species (ROS) scavenging enzymes) were found to be up-regulated by 4.00 fold and 3.72 fold in *L. casei* Zhang cultured with SSF-SW medium. In the cytoplasm, iron homeostasis can maintain intracellular “free” ferrous irons at a low concentration to limit Fenton reaction. In low-G + C-content gram-positive bacterium, the transcriptional regulator Spx is a major and highly conserved oxidative stress regulator. Moreover, MarR family transcriptional regulator can exert a global regulatory role on oxidative stress response by cysteine oxidation. The up-regulation of these three genes in *L. casei* Zhang cultured with SSF-SW medium was similar to that in *L. plantarum* CAUH2 in response to H_2_O_2_ stress [37]. In *Streptococcus mutans*, trehalose operon repressor TreR, which was up-regulated in *L. casei* Zhang cultured with SSF-SW medium, not only acted as the local regulator to govern trehalose utilization, but was also related to response to H_2_O_2_ stress [43].

Relative to the genes in *L. casei* Zhang cultured with LSF-MRS medium, in *L. casei* Zhang cultured with SSF-SW medium, seven genes encoding alcohol dehydrogenase (Adh), cold-shock protein, TetR/AcrR family transcriptional regulator, and DeoR/GlpR transcriptional regulator related to ethanol stress-tolerance were 2.12-fold to 19.93-fold up-regulated, respectively. The high up-regulation of alcohol dehydrogenases catalyzed the reversible reaction between ethanol and acetaldehyde, which might play important roles in detoxifying ethanol. Meanwhile, TetR/AcrR family regulator AcrR in *L. plantarum* NF92 was reported to participate in sorbitol or mannitol utilization by up-regulating the aldehyde-alcohol dehydrogenase encoding gene *adhE* [44]. In *L. plantarum,* the cold shock proteins acting as chaperons protected peptide synthesis from cold stress, and they were adapted to ethanol stress by down-regulating the transcriptional factor ctsR [45].

In comparison with *L. casei* Zhang cultured with LSF-MRS medium, in *L. casei* Zhang cultured with SSF-SW medium, the base excision repair system and homologous recombination were activated to relieve the DNA damage caused by adverse stress, which was consistent with the response of *L. plantarum* CAUH2 to adverse stress [37]. In base excision repair system, the DNA/RNA non-specific endonuclease and the uracil-DNA glycosylase were 2.94- and 2.10-fold up-regulated. In addition, for homologous recombination system, the DNA polymerase III subunit alpha DnaE and DNA repair protein RecF, RadC, and RadA were 2.11- to 4.22-fold up-regulated.

In *G. intestinalis*, some hypothetical proteins with Trx-like domains were significantly up-/down- regulated in response to oxidative stress, indicating that hypothetical proteins might play important roles in response to stress [42]. Compared to those cultured with LSF-MRS medium, in *L. casei* Zhang cultured with SSF-SW medium, the expression of six hypothetical proteins (LCAZH_0096, LCAZH_0137, LCAZH_1915, LCAZH_1916, LCAZH_2327, and LCAZH_2630) were up-regulated by 10.95 to 76.21 fold, which might be associated with the regulation of stress-tolerance, and the new functional proteins remains to be further explored in the future.

### 3.10. The Contents of Lactate and Acetate of L. casei Zhang after Fermentation with SSF-SW and LSF-MRS Media

**Figure 5:**
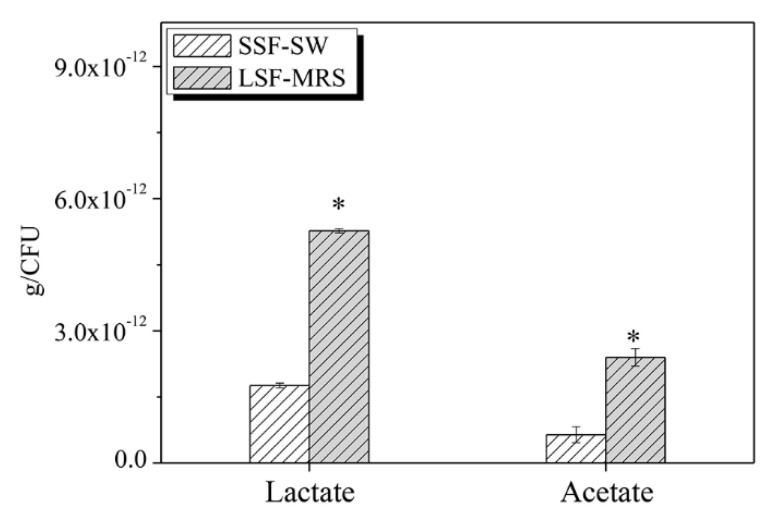
The contents of lactate and acetate of *L. casei* Zhang after fermentation with solid-state fermentation (in SSF-SW medium) and liquid-state fermentation (in LSF-MRS medium). * Statistically significant differences were determined by Student’s *t*-test.

For lactate, the total yield of *L. casei* Zhang fermented with LSF-MRS medium was found to be 5.27 × 10^−12^ ± 4.55 × 10^−14^ g/CFU, which was significantly higher than that fermented with SSF-SW medium (2.40 × 10^−12^ ± 1.97 × 10^−13^ g/CFU) (*p*-value < 0.05). Similarly, for acetate, the total yield of *L. casei* Zhang fermented with LSF-MRS medium was found to be 1.76 × 10^−12^ ± 5.41 × 10^−14^ g/CFU, which was significantly higher than that fermented with SSF-SW medium (6.42 × 10^−13^ ± 1.82 × 10^−13^ g/CFU) (*p*-value < 0.05) (Figure 5). The productions of lactate and acetate of *L. casei* Zhang fermented with SSF-SW medium were less than those of cells fermented with LSF-MRS medium, which seems to be the opposite of the transcriptome result. This may be because the SSF-SW medium is composed of bran and soybean meal, which is more difficult to use, so there may be fewer raw materials for acid production than LSF-MRS medium.

### 3.11. Total Biomasses of L. casei Zhang after Fermentation with SSF-SW Medium Supplemented with Different Carbon Sources

**Figure 6:**
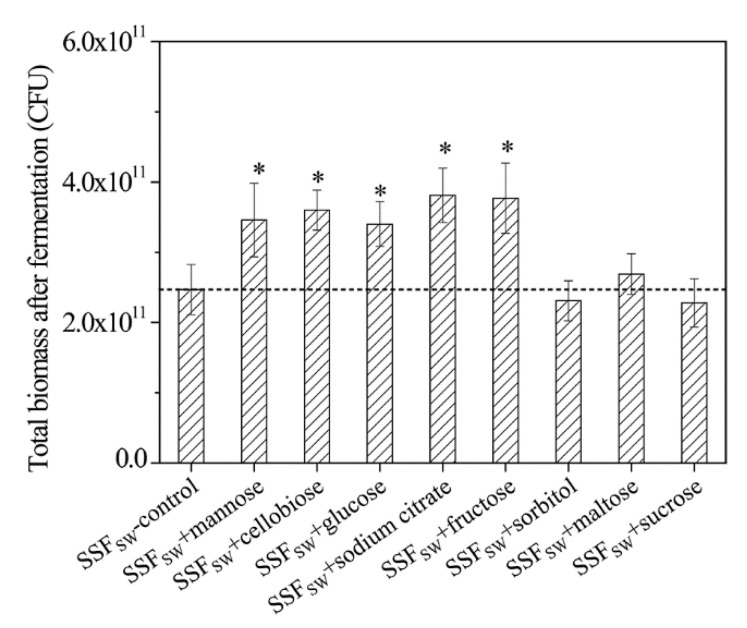
Total biomass of *L. casei* Zhang fermented with SSF-SW medium supplemented with different carbon sources. Supplementation amount was 1.0 wt %. * Statistically significant differences were determined by Student’s *t*-test.

As mentioned above, the stress-tolerance of *L. casei* Zhang after fermentation with SSF-SW medium was obviously higher than that after fermentation with LSF-MRS medium. However, the biomass of *L. casei* Zhang after fermentation with LSF-MRS medium was 2.24 times as much as that after fermentation with SSF-SW medium. In addition, the nutrient content of SSF-SW medium was indeed lower than that of LSF-MRS medium. Interestingly, compared to that after fermentation with LSF-MRS medium, the carbohydrate transport of *L. casei* Zhang after fermentation with SSF-SW medium was greatly enhanced. Therefore, we speculated that the supplementation with carbon source whose transport-related genes were up-regulated into medium would enhance the total biomasses of *L. casei* Zhang after fermentation with SSF-SW medium. As expected, compared to the blank control, after supplementation with 1.0% (wt %) of mannose, cellobiose, glucose, citrate, and fructose, the total biomass of *L. casei* Zhang after fermentation with SSF-SW medium was significantly increased by 40.08%, 45.75%, 37.65%, 54.25%, and 52.63%, respectively (Figure 6). Surprisingly, compared to the blank control, after supplementation with 1.0% (wt %) of sorbitol, maltose, and sucrose, the total biomass of *L. casei* Zhang after fermentation with SSF-SW medium exhibited no significant difference, which might be due to the unknown regulatory mechanisms. The maximum total biomass of *L. casei* Zhang fermented with SSF-SW medium (3.81 × 10^11^ CFU) was observed after supplementation with 1.0% (wt %) of citrate, which was still lower than that of *L. casei* Zhang fermented with LSF-MRS medium (6.01 × 10^11^ CFU). The medium composition and saccharides supplementation amount remain to be further explored in future research.

## 4. Conclusions

The NaCl, H_2_O_2_, and ethanol stress tolerance and the bacterial survival rate after bacterial agent preparation of *L. casei* Zhang were significantly improved after fermentation with SSF-SW medium, relative to fermentation with LSF-MRS medium. However, the biomass of *L. casei* Zhang fermented with SSF-SW medium was lower than that when fermented with LSF-MRS medium. Fortunately, mannose, cellobiose, glucose, citrate, and fructose were found to promote the total biomass of *L. casei* Zhang fermented with SSF-SW medium. The global transcriptomic analysis revealed a total of 1106 differentially expressed genes. By comparing LSF-MRS fermentation and SSF-SW fermentation, we found the changes in the following biological processes. Carbohydrate transport and gluconeogenesis were enhanced, and pentose phosphate pathway was up-regulated to produce more NADPH. Inositol phosphate metabolism was enhanced to produce acetyl-CoA and DAHP, citrate transport and fermentation was extremely up-regulated to produce more pyruvate and ATP, and pyruvate metabolism was widely up-regulated to generate more lactate, acetate, ethanol, and succinate. However, glycolysis was suppressed, and fatty acid biosynthesis was also suppressed to decrease the utilization of acetyl-CoA. Some stress tolerance-related genes which encoded aquaporins (GlpF), superoxide dismutase (SOD), nitroreductase, four iron homeostasis-related proteins, trehalose operon repressor TreR, alcohol dehydrogenase (ADH), two TetR/AcrR family transcriptional regulators, and six hypothetical proteins, were found to be up-regulated in *L. casei* Zhang after fermentation with SSF-SW medium. Our findings provide novel insight into the differences in *L. casei* Zhang from solid-state and liquid-state fermentations and prove that the culture medium can enhance the resistance of food starter to certain environmental conditions. This study will be useful for improvement of bacterial stress tolerance and bacterial agent biomass in fermented food and feeding industry. In addition, future works will evaluate the resistance of lactic acid bacteria to acid, heat, and antibiotic stress after solid-state fermentation, and a deeper investigation of the metabolites produced after solid-state fermentation by *L. casei* Zhang is necessary.

## Supplementary Materials

The following are available online at https://www.mdpi.com/2076-2607/8/11/1656/s1, Figure S1: Pearson correlation between samples; Figure S2: Principal component analysis (PCA) between samples; Figure S3: DEGs in *L. casei* Zhang after fermentation with solid-state fermentation (in SSF-SW medium) and liquid-state fermentation (in LSF-MRS media) by GO enrichment; Figure S4: DEGs in *L. casei* Zhang after fermentation with solid-state fermentation (in SSF-SW medium) and liquid-state fermentation (in LSF-MRS medium) by KEGG pathway enrichment, Table S1: The number of reads before and after the trimming step; Table S2: The information of reads mapping to the reference genome and genes; Table S3: Genes differentially expressed at the transcriptional level in *L. casei* Zhang after fermentation with solid-state fermentation (in SSF-SW medium) and liquid-state fermentation (in LSF-MRS medium).
