# Supplementary material for: Differential Analysis of Stress Tolerance and Transcriptome of Probiotic Lacticaseibacillus casei Zhang Produced from Solid-State (SSF-SW) and Liquid-State (LSF-MRS) Fermentations
Source: Microorganisms. 2020 Oct 26;8(11):1656. doi: 10.3390/microorganisms8111656 (PMC7716342; doi:10.3390/microorganisms8111656)
Supplement: Supplementary file 1 [file microorganisms-08-01656-s001.zip › Supplementary materials/Supplementary Material.docx]

**Supplementary Material**

**Differential Analysis of** **Stress tolerance and Transcriptome of** **Probiotic *Lacticaseibacillus casei* Zhang produced from Solid-state (SSF-SW) and Liquid-state (LSF-MRS) Fermentations**

Table S1 The number of reads before and after the trimming step.

| Samples | Total raw reads  (M) | Total clean reads  (M) | Total clean bases  (Gb) |
| --- | --- | --- | --- |
| SSF-SW-1 | 21.93 | 21.45 | 1.07 |
| SSF-SW-2 | 21.93 | 21.5 | 1.07 |
| SSF-SW-3 | 21.94 | 21.56 | 1.08 |
| LSF-MRS-1 | 21.94 | 21.48 | 1.07 |
| LSF-MRS-2 | 21.94 | 21.46 | 1.07 |
| LSF-MRS-3 | 21.94 | 21.45 | 1.07 |

Table S2 The information of reads mapping to the reference genome and genes.

| Samples | Mapping to the reference genome | | |  | Mapping to the reference genes | | |
| --- | --- | --- | --- | --- | --- | --- | --- |
|  | Total  clean reads  (M) | Total  mapping  (%) | Uniquely  mapping  (%) |  | Total  clean reads  (M) | Total  mapping  (%) | Uniquely  mapping  (%) |
| SSF-SW-1 | 21.45 | 99.19 | 96.77 |  | 21.45 | 75.95 | 75.57 |
| SSF-SW-2 | 21.50 | 99.23 | 97.16 |  | 21.50 | 76.05 | 75.75 |
| SSF-SW-3 | 21.56 | 99.35 | 97.80 |  | 21.56 | 76.63 | 76.28 |
| LSF-MRS-1 | 21.48 | 99.44 | 98.30 |  | 21.48 | 79.56 | 79.23 |
| LSF-MRS-2 | 21.46 | 99.51 | 98.29 |  | 21.46 | 77.02 | 76.66 |
| LSF-MRS-3 | 21.45 | 99.42 | 98.36 |  | 21.45 | 78.37 | 78.04 |


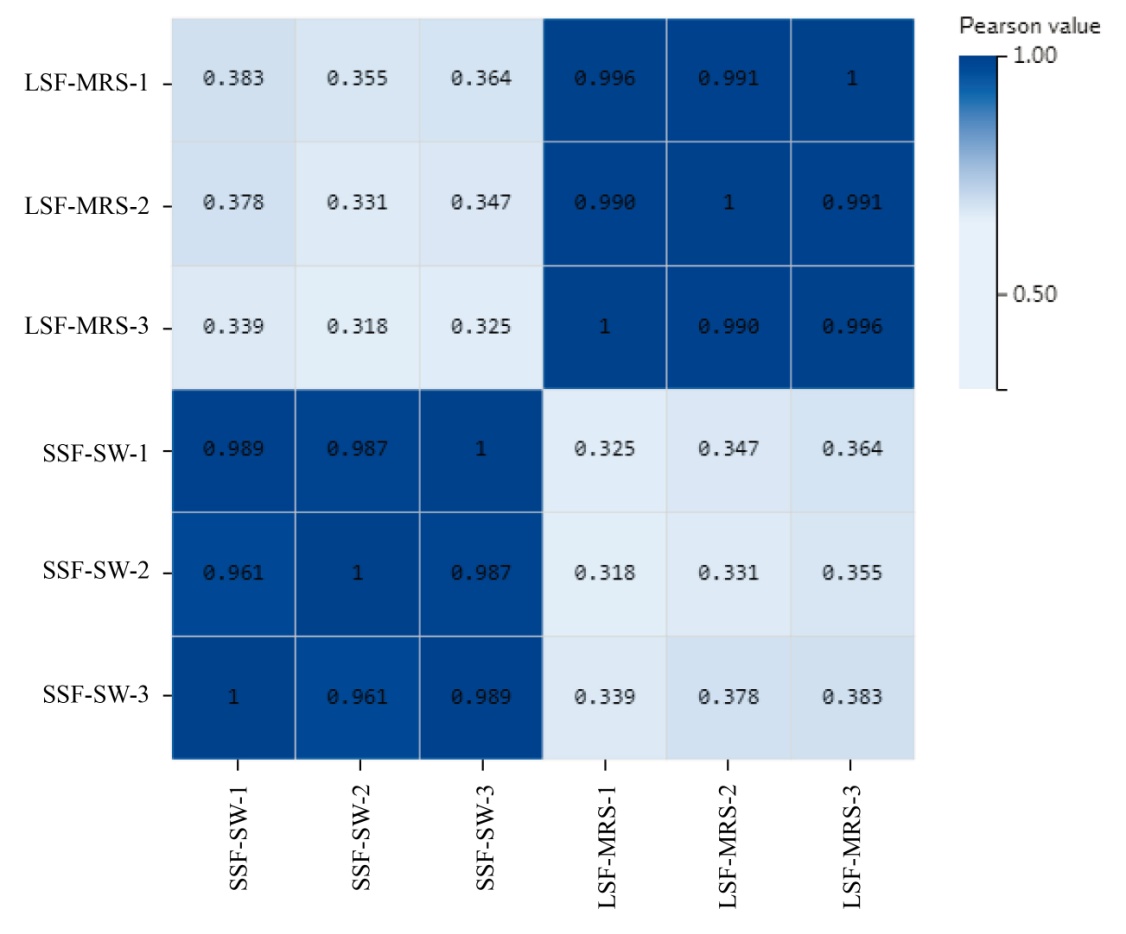


**Fig. S1** Pearson correlation between samples.


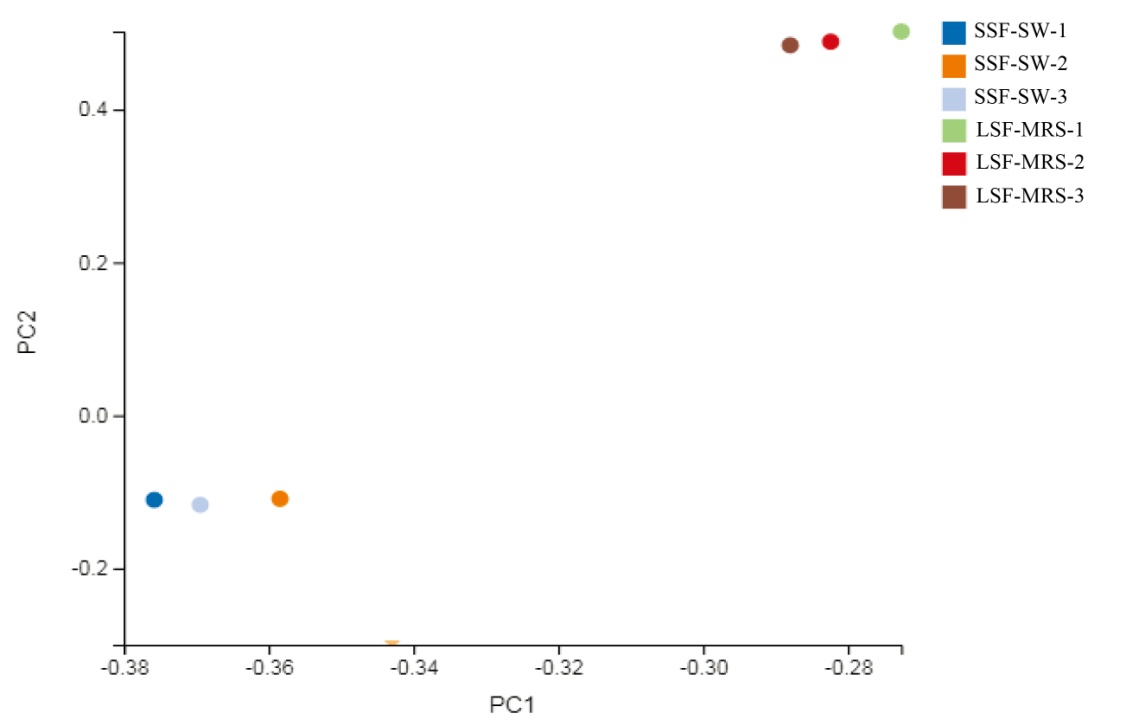


**Fig. S2** Principal component analysis (PCA) between samples.


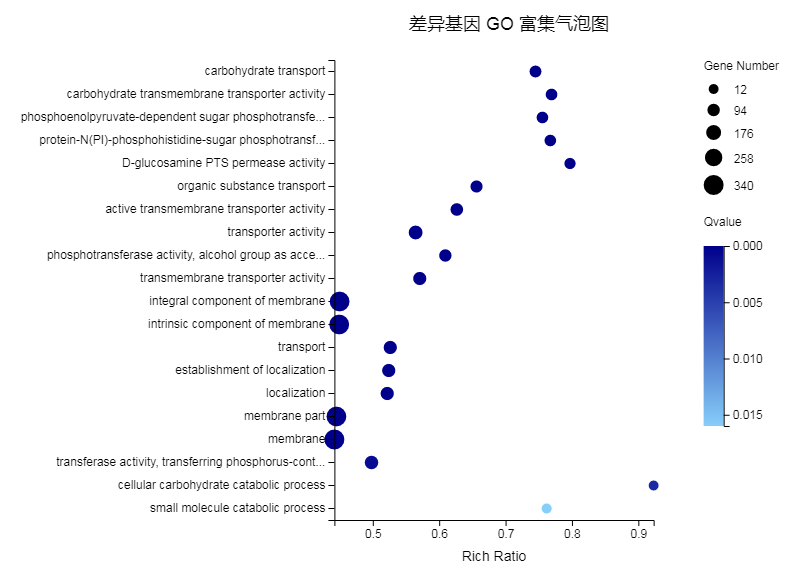


**Fig. S3** DEGs in *L. casei* Zhang after fermentation with solid-state fermentation (in SSF-SW medium) and liquid-state fermentation (in LSF-MRS media) by GO enrichment.


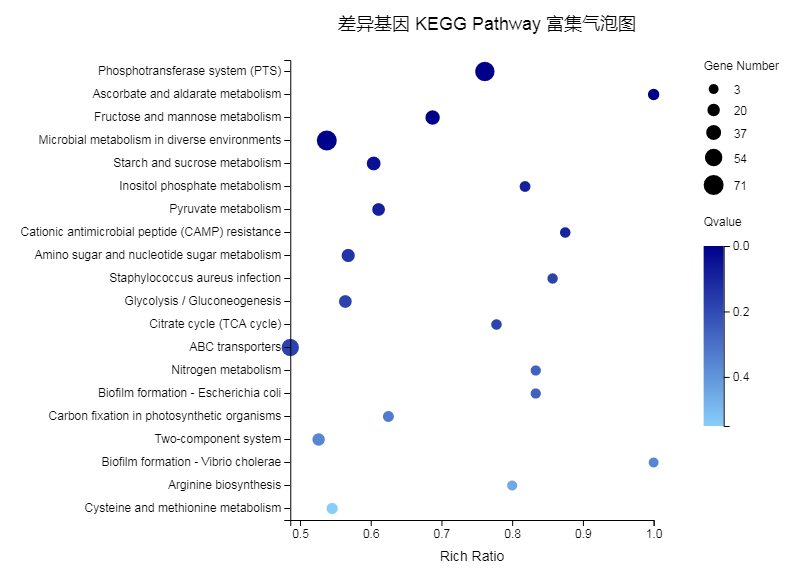


**Fig. S4** DEGs in *L. casei* Zhang after fermentation with solid-state fermentation (in SSF-SW medium) and liquid-state fermentation (in LSF-MRS medium) by KEGG pathway enrichment.
